# Trunk control and acute-phase multifactorial predictors of community mobility after stroke: a longitudinal observational study

**DOI:** 10.3389/fneur.2024.1376444

**Published:** 2024-04-24

**Authors:** Randah Ahmed Alomari, Ejlal Abdullah BinMulayh, Abdullah Mohammad Alqarni, Mashael Alsobhi, Mohamed Faisal Chevidikunnan, Reem Basuodan, Fayaz Khan

**Affiliations:** ^1^Department of Physical Therapy, Faculty of Medical Rehabilitation Sciences, King Abdulaziz University, Jeddah, Saudi Arabia; ^2^Ministry of Health, Abha Health Cluster, Abha, Saudi Arabia; ^3^Department of Rehabilitation Sciences, College of Health and Rehabilitation Sciences, Princess Nourah Bint Abdulrahman University, Riyadh, Saudi Arabia

**Keywords:** community mobility, trunk control, stroke, acute, moderation, prediction

## Abstract

**Introduction:**

Stroke is a detrimental condition associated with long-term functional impairments that restrict community reintegration, which is an indicator of successful post-stroke functional recovery and rehabilitation. Additionally, trunk control is an understudied factor that may contribute to community mobility and participation after stroke. This study aimed to identify predictors of community mobility among stroke survivors in the acute phase, with a primary focus on trunk control, in addition to exploring the mediating and moderating role of predictive factors.

**Methods:**

A longitudinal observational study included 61 participants with acute stroke. Trunk control test (TCT) during sitting, stroke severity, quality of life, fear of falls, depression, and age was assessed during the acute phase as potential predictors. The community mobility outcome measure was assessed 3 months after baseline using the Reintegration to Normal Living Index (RNLI). Statistical analyses included correlation, linear regression, mediation, and moderation analyses.

**Results:**

Trunk control test was the strongest predictor of RNLI among all factors (β = 0.72; 95%CI = 0.004–0.007; *p* ≤ 0.0001). Stroke severity, quality of life, fear of falls, and age significantly predicted RNLI (*p* < 0.01). Higher age was a significant moderator of the relationship between TCT and RNLI (β = 0.002; *p* < 0.001; 95% CI = 0.0001–0.0003).

**Discussion:**

The findings highlight sitting trunk control impairment during the acute stage as a crucial predictor of reduced community mobility after stroke, where age over 60 years can moderate this relationship. The study emphasizes that addressing trunk control during early stroke rehabilitation may enhance community reintegration prospects.

## 1 Introduction

Stroke can be detrimental due to long-term disability (1, 2), hindering functional performance and quality of life (QOL) (3). Physical and cognitive deficits associated with stroke limit daily activities and community participation (4). Based on the International Classification of Functioning, Disability, and Health (ICF) model, the domains affected by a health condition include body structures and functions (impairments), individual activities, and participation (denoting functional performance at the societal level) (4–6). Stroke rehabilitation approaches focus primarily on body function and individual activity domains (7), presuming it will eventually improve participation (8). However, such community participation improvements are not guaranteed (4).

Most stroke survivors experience long-term participation restrictions and consider it an unmet need (9–12), which is related to patient satisfaction after stroke (13). Even individuals with a mild stroke can have reduced participation in meaningful activities, such as work-related activities, volunteering, traveling, and socializing (14). In addition, some patients with stroke experience limited community ambulation even when they show good mobility levels on standardized measures (15). Community ambulation was reported as an essential or very important ability for the majority of stroke patients (15), which is only achieved by less than 50% of stroke survivors (16), ensuring that community participation is a meaningful outcome (15), and there is a need for developing rehabilitation approaches to enhance community participation after stroke (17). One proposed indicator of successful stroke rehabilitation is when a patient’s functional recovery enables them to achieve pre-stroke community participation (18).

A critical aspect of participation is community mobility, which refers to the functional abilities required to navigate and access transportation, the community environment, and public facilities, whether it can be achieved by ambulation or other forms of transferring, such as wheelchairs, cycling, or driving (19). Hence, while ambulation is a component of community mobility, it does not fully capture the complexity of the community mobility concept (19). Additionally, even though poor community mobility can persist up to 4 years after stroke, there remains a paucity of literature on community mobility predictors and outcomes after stroke (19). Generally, limited participation in the community after stroke has been associated with older age (11), stroke severity (20), reduced physical (21) and cognitive function (22), and depressive symptoms (12). Moreover, gait velocity, walking endurance, balance, and motor function can discriminate between different levels of the ability to ambulate at home and within the community (15, 23–25). Furthermore, social support was correlated with community participation after stroke as a strong predictor (17), but functional limitations correlated more strongly with community participation outcomes (4, 26, 27).

Community mobility is impacted by physical functioning (19), and achieving functional independence increases the likelihood of community participation after a stroke (17). Accordingly, addressing how factors correlated with functional outcomes could contribute to community mobility outcomes can provide helpful insights into understanding limited community mobility after stroke. For instance, trunk control has been identified as a critical early predictor of post-stroke functional outcomes (28, 29), positively correlated with stroke patients’ capability of postural control, ambulation, and functional activities in the chronic stage (30, 31). Furthermore, measures of sitting trunk control during the first 2 weeks of the acute phase after stroke can predict the likelihood of restoring independent walking after 3 months (25, 32). Nevertheless, no previous studies examined trunk control as a predictor of post-stroke community mobility and participation.

Predicting stroke outcomes can provide valuable insights for patients and healthcare providers (33). Particularly, outcomes related to long-term disability can be of more value when addressed during the acute phase to guide rehabilitation programs and maximize independence and QOL after stroke (34, 35). Therefore, the current study aimed to identify potential factors measured during the acute phase, primarily trunk control that can predict community mobility among stroke survivors. Additionally, a secondary aim of the study is to explore the mediating and moderating effects of factors, including age, stroke severity, QOL, anxiety, depression, and fear of falls, on the relationship between trunk control and community mobility. The primary hypothesis is that trunk control, as measured during the acute phase of stroke, will be a significant predictor of community mobility among stroke survivors.

## 2 Materials and methods

### 2.1 Study design

A prospective longitudinal observational study design was used to address community mobility outcomes and to help understand temporal relationships, i.e., whether potential predictors precede community mobility outcomes. Data were collected from January to June 2022 at three major hospitals affiliated with the Ministry of Health. Ethical approval was obtained from the Institutional Review Board of the Ministry of Health (A01249), which is approved by the National Committee of Bioethics (NCBE-KACST, KSA: H-02-J-002).

### 2.2 Participants

Sixty-one out of 140 participants met the eligibility criteria and were included in the study through consecutive sampling. The sample size was calculated using G power version 3.1, where the value for alpha was set at 0.05, 1-beta at 0.8, and effect size at 0.15 for regression based on Cohen’s medium effect size for multiple correlations (36). Since the study included six predicting factors, a minimum sample of 60 participants (10 participants for each factor) was determined to achieve 80% power to detect statistical significance in the community mobility outcome measure (37). The study included participants aged 18 years and older who had been diagnosed with stroke within 1 month prior to enrollment, as confirmed by computed tomography (CT) or magnetic resonance imaging (MRI) scans. This timeframe was used to ensure that the participants were in the acute phase of stroke. Participants were excluded from the study if they had severe comorbidities such as advanced renal disease, active malignancy, or acute systemic infections that could impact recovery, in addition to patients with brainstem stroke or bilateral stroke. Furthermore, individuals unable to comprehend and follow instructions were excluded to ensure adequate participation in the study assessments.

### 2.3 Outcome measures

The trunk control was measured by the trunk control test (TCT), which includes assessment items of rolling to both sides (two items; one for each side), sitting position (one item), and maintaining sitting balance (one item) on a three-point ordinal scale (0 = unable to perform without assistance; 12 = able to perform with external assistance or in an atypical manner; 25 = able to perform normally) (38). The total score ranges from 0 to 100 (a score of 100 is achieved by getting 25 points on each of the four items), where higher scores indicate better performance (38). As reported by previous studies, TCT has excellent interrater reliability and predictive validity in the stroke population (38–40).

Stroke severity was measured by the modified Rankin Scale (mRS), a measure of global disability and recovery after stroke, which has excellent interrater, intra-rater, and test–retest reliability in acute stroke (17, 41).

Quality of life (QOL) was measured using the Stroke-Specific QOL Scale (SSQOL), commonly used to provide a comprehensive evaluation of QOL in patients with stroke (42). The scale consists of 49 questions rated on a five-point Likert scale, addressing 12 domains: mobility, upper limb functions, vision, social role, self-care, family role, work-productivity, language, energy, mood, personality, and thinking (42). The overall score of the SSQOL scale has excellent construct validity in the acute stroke population (43).

The Hospital Anxiety and Depression Scale (HADS) assessed anxiety and depressive symptoms (44). HADS consists of two subscales for anxiety and depression, seven items each, scaled from 0 to 3 as symptoms increase; it showed good reliability for both subscales and excellent construct validity in acute stroke (44). A score higher than 8 can be indicative of depression in acute stroke (45).

Fear of falls was measured using the Modified Falls Efficacy Scale (MFES), which addresses the level of patients’ confidence in performing 14 daily activities independently from 0 (not confident at all) to 10 (completely confident), which has excellent test–retest reliability (46).

An indication of received social support was measured by a yes/no answer to whether the participant often has a family member or friend whom they can rely on for accompaniment to appointments, social activities, or community engagements outside their household.

Community mobility was assessed as a dependent variable using the Reintegration to Normal Living Index (RNLI), a self-reported outcome of 11 items addressing physical, social, and psychological aspects of community reintegration, such as community mobility, recreational and work-related activities, the degree of comfort with one’s role in the family and personal relationships, and the ability to manage life events (47, 48). The RLNI visual analog scale with a normalized score of 100 was used in this study (a higher score represents more community integration) (47, 49). The RNLI has excellent internal consistency, moderate test–retest reliability, and adequate construct validity in the chronic stroke population (50–52).

### 2.4 Procedures

**Figure 1:**
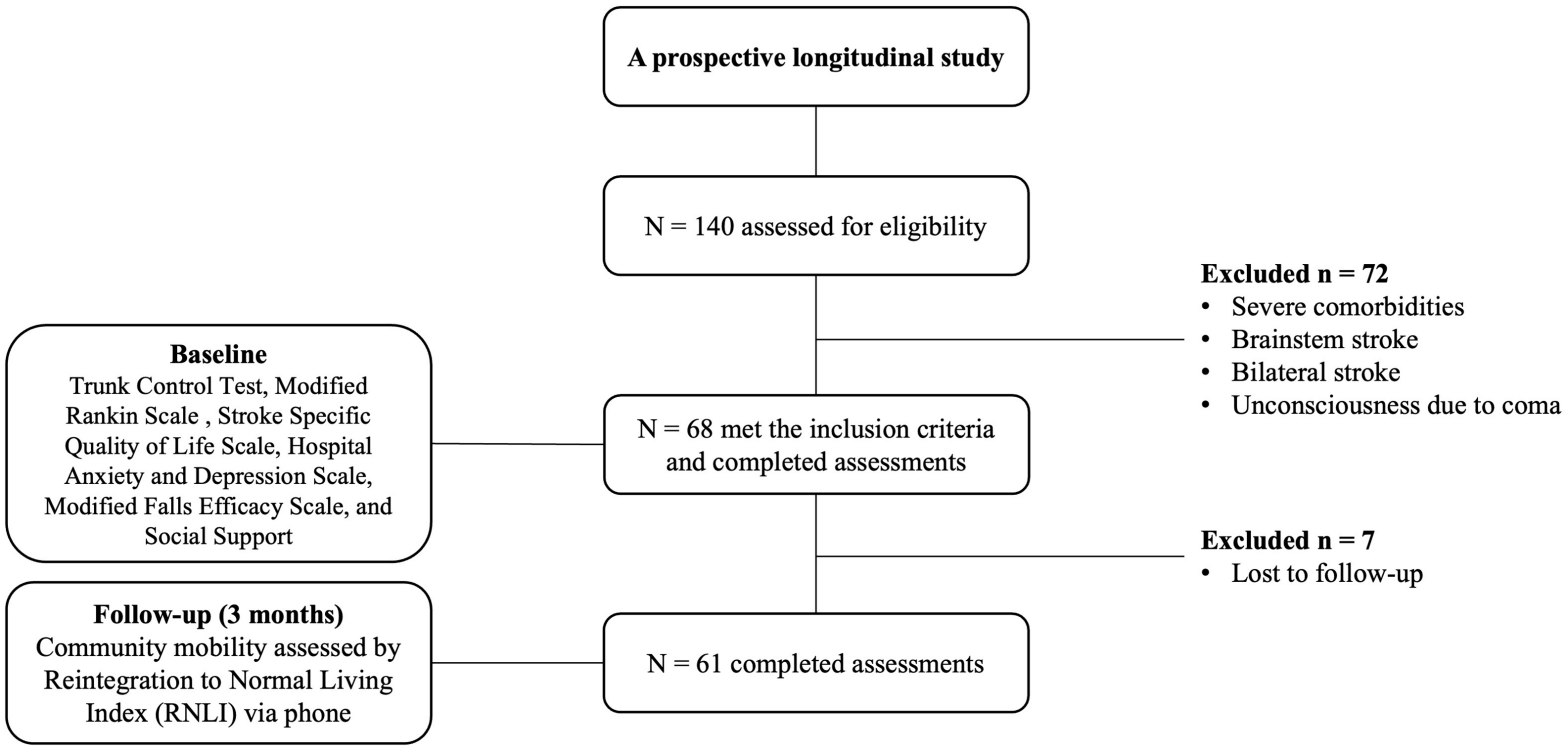
Study design and participants’ flow chart.

Research physical therapists were assigned to the three hospitals, and they visited the inpatient wards of the stroke unit and internal medicine department where patients with acute stroke were admitted. The in-charge nurses informed the research physical therapists about new admissions and handled the consent form process with patients who agreed to participate in the study. At baseline during the first visit, data on social and demographic characteristics were initially collected, including age, gender, marital status, social support, stroke type, stroke site, stroke duration, and hemiplegic side. Assessments of trunk control, resilience, stroke severity, QOL, depression, and fear of falls were administered by a trained physical therapist during the acute phase in the inpatient setting. All the assessments were performed in the inpatient setting by the same physical therapists, who also evaluated the follow-ups. During the follow-up period, study physical therapists made monthly calls to participants or caregivers, confirming availability for follow-up phone assessment and gathering updates on community mobility and barriers. After 3 months of the baseline assessment, telephone-based data collection was used for community mobility. The RNLI has been validated for collection using telephone interview mode (49, 53), allowing for cost-effective and efficient follow-up data collection among our participants and research personnel (Figure 1).

### 2.5 Statistical analysis

Collected data were analyzed using IBM SPSS software for Windows, version 25 (IBM SPSS, IBM Corp., Armonk, N.Y., United States). Descriptive statistics were expressed as mean and standard deviation for normally distributed data and median and range for skewed data. Pearson’s correlation was performed to assess the correlation between community mobility (i.e., RNLI) and the other outcome measures (i.e., TCT, BRS, mRS, SSQOL, HADS, and MFES). Simple linear regression was conducted to examine the effect of each outcome measure on community mobility (i.e., RNLI) as a dependent variable, followed by multiple linear regression to find the best predictor for community mobility. The assumptions for multiple linear regression were tested, and the variables did not assume normality. Hence, to overcome that, bootstrapping was performed. The variance inflation factor (VIF) was checked for multicollinearity, and it was less than 5 for all the variables. Heteroscedasticity was tested with the assumption of the Breusch–Pagan test (*p* ≥ 0.05). Mediation and moderation analyses were conducted using Hayes PROCESS to identify potential mediation and moderation of the outcome measures on community mobility. The level of significance was set at a *p* value of ≤0.05 for all the analyses.

## 3 Results

### 3.1 Demographic and baseline characteristics

A total of 61 participants, with a mean age of 58 ± 12.5 years, were included in the study analysis. The baseline demographic characteristics of the participants are presented in Table 1.

**Table 1:** Demographic and baseline characteristics of participants.

Age – mean ± SD (range)	58 ± 12.5 (28–80)
Gender—*n* (%)	
*Male*	37 (60.7)
*Female*	24 (39.3)
Marital status—*n* (%)	
*Married*	54 (88.5)
*Single*	7 (11.5)
Living place—*n* (%)	
*Urban*	55 (90.2)
*Rural*	6 (9.8)
Social support—*n* (%)	
*Yes*	56 (91.8)
*No*	5 (8.1)
Stoke side—*n* (%)	
*Right*	33 (54.1)
*Left*	28 (45.9)
Stroke type—*n* (%)	
*Ischemic*	41 (67.2)
*Hemorrhagic*	20 (32.8)
Stroke duration—mean ± SD (range)	8.7 ± 8 (1–30)
Outcome measures—median (range)	
*TCT*	66 (0–100)
*mRS*	2.7 (0–5)
*SSQOL*	149.5 (56–242)
*HADS depression*	7.2 (0–19)
*HADS anxiety*	6.6 (1–20)
*MFES*	5.6 (0–10)
*RNLI*	0.68 (0–1)

SD, Standard deviation; TCT, Trunk control test, mRS, Modified Rankin scale; SSQL, Stroke-specific quality of life; HADS, Hospital anxiety and depression scale; MFES, Modified fall efficacy scale; and RNLI, Reintegration to normal living index.

### 3.2 Correlations between community mobility and other outcome measures

The correlation analysis used the dependent variable of community mobility measured by RNLI. There was a significant positive correlation between RLNI and each of TCT (*r* = 0.72; 95% CI = 0.59–0.83; *p* value < 0.0001), SSQOL (*r* = 0.59; 95% CI = 0.37–0.72; *p* value <0.0001), and MFES (*r* = 0.47; 95% CI = 0.26–0.65; *p* value <0.0001). Additionally, there was a significant negative correlation between RLNI and each of mRS (*r* = −0.64; 95% CI = −0.78 to −0.49; *p* value <0.0001), and HADS depression (*r* = −0.31; 95% CI = −0.53 to −0.08; *p* value = 0.01). The correlation coefficient values among all independent variables were checked in a correlation matrix to control for multicollinearity, which was less than 0.7, and the VIF value was less than 1.5 in multiple regression.

### 3.3 Factors predicting community mobility—simple linear regression

Simple linear regression analysis was performed using RLNI score after 3 months post-stroke as the dependent variable to determine the influence of each outcome measure on community mobility levels after 3 months. The significant predicting factors were trunk control measured by TCT (β = 0.77), stroke severity measured by mRS (β = −0.66), QOL measured by SSQOL (β = 0.57), fear of fall measured by MFES (β = 0.46), and age (β = −0.36).

**Table 2:** Multiple linear regression with bootstrapping results for study variables.

									Bootstrap	
	Unstandardized coefficient		Standardized coefficient		95% CI			95% CI		
Independent variables	B	SE	Beta	*t*	Lower bound	Upper bound	*p* value	Lower bound	Upper bound	*p* value
*TCT*	0.005	0.001	0.562	3.508	0.002	0.007	0.001	0.002	0.007	0.006
*Age*	−0.003	0.002	−0.114	−1.148	−0.008	0.002	0.256	−0.009	0.006	0.478
*Mrs*	−0.035	0.037	−0.186	−0.939	−0.110	0.040	0.352	−0.099	0.030	0.303
*SSQL*	0.001	0.001	0.042	0.251	−0.002	0.003	0.802	−0.003	0.004	0.825
*MFES*	−0.006	0.011	−0.074	−0.547	−0.029	0.016	0.587	−0.027	0.019	0.603
*HADS depression*	−0.008	0.007	−0.140	−1.114	−0.023	0.007	0.270	−0.024	0.008	0.282
*HADS anxiety*	0.013	0.006	0.221	2.039	0.001	0.025	0.047	0.002	0.022	0.041

SE, Standard error; 95% CI, 95% confidence interval; TCT, Trunk control test, mRS, Modified Rankin scale; SSQL, Stroke-specific quality of life; MFES, Modified fall efficacy scale; HADS, Hospital anxiety and depression scale; and BRS, Brief resilience scale.

### 3.4 Factors predicting community mobility—multiple linear regression

A multiple linear regression analysis was performed to identify the best predictor for community mobility among the significant predicting factors (Table 2). Trunk control assessed by TCT was identified as the strongest predictor of post-stroke community mobility among all factors (β = 0.72; 95% CI = 0.004–0.007; *p* value <0.0001) along with HADS Anxiety (β = 0.22; 95% CI = 0.001–0.025; *p* value = 0.047). The goodness of fit for the model for ANOVA was *p* ≤ 0.0001, *F* = 10.076 and adjusted *R*^2^ = 0.52.

### 3.5 Mediation and moderation analyses

**Figure 2:**
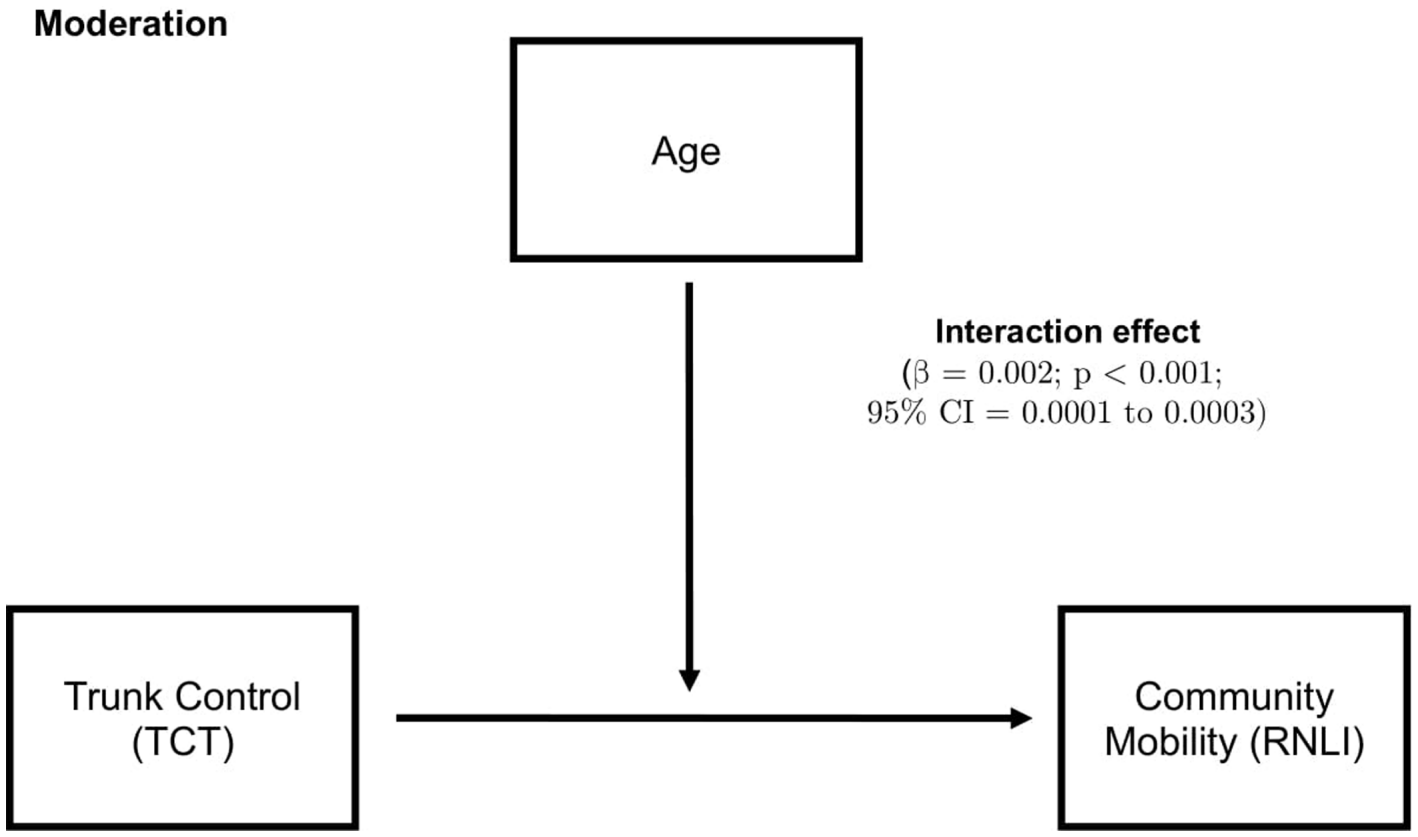
Moderation analysis, where higher age moderates the relationship between trunk control and community mobility. 95% CI, 95% confidence interval; TCT, Trunk control test; and RNLI, Reintegration to normal living index.

The model that included age as a moderator, as demonstrated in Figure 2, showed that age significantly moderates the relationship between TCT and community mobility (β = 0.002; *p* < 0.001; 95% CI = 0.0001–0.0003). The conditional effect of age on community mobility showed that at low moderation, age = 43, (conditional effect = 0.0018; *p* = 0.107; 95% CI = −0.0004–0.0041). At middle moderation, age = 60 (conditional effect = 0.0053; *p* < 0.0001; 95% CI = 0.004–0.007). At high moderation, age = 73, (conditional effect = 0.0081; *p* < 0.0001; 95% CI = 0.006–0.01). There was no significant result for the mediation analysis.

## 4 Discussion

The present study was undertaken to address potential factors that can serve as predictive indicators of community mobility among stroke survivors based on outcome measures obtained during the acute phase, where trunk control was the primary outcome measure. Further, the study investigated whether age could mediate or moderate the relationship between trunk control and community mobility.

Our results showed that higher trunk control, better QOL, less fear of falls, lower stroke severity, and fewer depressive symptoms in the acute phase were significantly correlated with more community reintegration after stroke. Additionally, trunk control, stroke severity, QOL, fear of falls, and age were the significant factors that can predict community mobility after stroke. Among these significant factors, trunk control was the strongest predictor of post-stroke community mobility, and anxiety was the second strongest predictor. Moreover, the findings highlighted that age significantly moderates the relationship between trunk control and community mobility.

Despite the reported limitations in community reintegration after stroke, the impact of specific post-stroke deficits on this ability remains undetermined (54). Relevant factors identified in previous studies aligned with the current results include stroke severity, aging, QOL, depressive symptoms, activities of daily living, fear of falls, and physical function and performance (17, 19, 54–57). Previous research has also established that extending rehabilitation intervention for longer than 6 months or 1 year after stroke can promote benefits in the physical aspects that are potentially associated with reduced community mobility, although the recovery of community integration in the later chronic stage of stroke is still not guaranteed (17, 19). The current findings, alternatively, identified trunk control in the acute stage of stroke as a strong predictor of community mobility, which has been highlighted previously to strongly predict the recovery of independent walking after stroke (32).

Trunk control during normal walking requires a coordinated movement between the upper and lower trunk, which is significantly altered during stroke-related pathologic gait (58), especially since trunk muscles are affected by the reduced level of activities and coordination after stroke (59). Trunk control allows shifting the body weight toward the non-paretic side or walking aid even with minimal voluntary movements of the lower limbs (60), and trunk lateral flexion and rotation combined with hip circumduction allow taking a step forward with the paretic side while walking (32). Besides, adequate trunk control is required to achieve most activities of daily living, such as bed mobility, sitting, standing, and transferring tasks (30).

A prior study used an algorithm based on classification and regression analysis to predict independent walking, performed by bedside assessments at 1 week following a stroke (32). Using TCT to assess trunk control, they found that patients with TCT scores higher than 40 at 1 week achieved independent walking after 6 weeks, whereas patients with TCT scores lower than 40 did not walk independently until 12 weeks post-stroke, suggesting that trunk control is a strong predictor of independent walking recovery after stroke (32). Another study conducted a randomized controlled trial and observed that interventions targeting trunk muscles in chronic stroke enhanced trunk control, mobility, and community reintegration more than standard physical therapy interventions (30). Similarly, a systematic review of trunk biomechanics after stroke highlighted that trunk deficits are critical for walking recovery, and trunk training should be integrated into post-stroke rehabilitation programs (58). The current findings similarly support the importance of trunk control as a strong predictor of community mobility 3 months after stroke, which further suggests that addressing trunk control early after stroke may influence not only independent walking but community reintegration as well. One possible explanation for this finding could be related to how functional trunk movements involve indirect cortical connections to the medial descending pathways of the extra-pyramidal system, including the vestibulospinal and reticulospinal tracts, which collectively have a potential contribution to improving sensory motor function, postural control, and dynamic reactive balance after stroke (30, 61). In fact, such abilities in motor coordination and dynamic balance have been shown to correlate with the level of community mobility that can be achieved after stroke (19).

The results also demonstrated the role of higher age in moderating the direct relationship between trunk control and community mobility, where the significant moderating effect of age was found at 60 years, and older ages were associated with a higher significance level. A prospective cohort study of stroke survivors reported that older adults experienced lower community reintegration levels than younger individuals (54). Similar findings in other studies reported that advanced age was associated with restricted post-stroke activity participation (62), community participation (63), return to work (55), and community mobility measured by the RNLI (64). Our findings also support age as a predicting factor for post-stroke community mobility. Alternatively, some studies found that age was not associated with community participation, and physical outcome measures had a stronger association (17, 65), although age can have a complex interaction with community and social participation (66). While the results in this study presented that trunk control was a stronger predictor of community mobility than age, it also described the interaction effect of age in moderating the relationship between trunk control as a physical outcome measure and community reintegration level after stroke.

Further factors identified by the present study in predicting post-stroke community mobility were anxiety, stroke severity, fear of falls, QOL, and depressive symptoms. A previous study concluded that the presence of anxiety in the acute phase of stroke is negatively associated with health-related QOL (67), which is a predictor for depression, and evidence suggest it should be screened routinely after stroke (68). Prior results also showed that stroke severity and dependent activities of daily living had a significant correlation with limited social participation (56). A cross-sectional study reported that restricted community mobility was related to fear of falling in individuals with chronic stroke (57). In a longitudinal study, stroke-related depressive symptoms were linked to reduced activity participation 1 year after stroke (12). Additionally, depressive symptoms can contribute to a lower QOL, which has been found to predispose stroke survivors to limitations in community mobility (19).

The present findings contribute to advancing our understanding of factors influencing community mobility after stroke, especially since stroke-related impairments directly impacting community reintegration are still not fully understood (54). Notably, identifying trunk control during the acute stage as a strong predictor of community mobility at 3 months post-stroke provides valuable insights, which underscore the potential value of emphasizing trunk control assessment and rehabilitation in early stroke recovery. By addressing trunk control deficits through targeted interventions during acute stroke rehabilitation, clinicians may enhance prospects for community reintegration. Moreover, the observed moderating effect of age suggests that older individuals may be particularly vulnerable to the impact of impaired trunk control on community mobility. Hence, addressing trunk control deficits among older stroke patients can be critical to optimizing their community mobility outcomes.

The current study has several limitations that need to be considered before interpreting the results. While the present study was conducted in Jeddah, Saudi Arabia, and most participants lived in urban areas, the findings may apply to stroke populations in similar settings within the region. However, caution should be exercised when generalizing the results to rural or substantially different cultural contexts, as various environmental and societal factors can influence community reintegration. Hence, the current results may not fully capture the diverse characteristics present in stroke populations across different geographical regions around the globe, restricting the generalizability of the findings to other regions and populations. Another limitation is that community mobility was assessed 3 months post-stroke through telephone interviews using the RNLI rather than in-person administration. Since the RNLI relies on self-reported data, the responses may have been subject to recall bias. Finally, although the longitudinal observational design allowed for examining factors over time, the nature of the study does not permit conclusions about causal relationships between community mobility and the identified predictors, such as trunk control. Future research using experimental methods may provide stronger evidence regarding causality.

In conclusion, this study found that trunk control during the acute stage of stroke was the strongest predictor of community reintegration levels 3 months post-stroke. Stroke severity, QOL, fear of falling, and patient age also emerged as significant predictive factors. Notably, higher age (60 years and above) moderated the relationship between trunk control and community mobility, where this relationship was more pronounced in older individuals. These findings underscore the potential impact of impaired trunk control in the acute phase on reduced community mobility after stroke. The results provide valuable insights highlighting the importance of assessing and addressing trunk control impairments through early stroke rehabilitation, particularly for older patients, suggesting that emphasizing trunk control in future studies and clinical interventions may enhance community reintegration prospects for stroke survivors.

## Data availability statement

The raw data supporting the conclusions of this article will be made available by the authors, without undue reservation.

## Ethics statement

The studies involving humans were approved by Institutional Review Board of the Ministry of Health (A01249), which is approved by the National Committee of Bioethics (NCBE-KACST, KSA: H-02-J-002). The studies were conducted in accordance with the local legislation and institutional requirements. The participants provided their written informed consent to participate in this study. Written informed consent was obtained from the individual(s) for the publication of any potentially identifiable images or data included in this article.

## Author contributions

RA: Writing – original draft, Methodology, Data curation, Conceptualization. EB: Writing – original draft, Validation, Methodology, Data curation. AA: Conceptualization, Formal analysis, Resources, Supervision, Writing – review & editing. MA: Writing – original draft, Validation, Resources, Methodology, Data curation, Conceptualization. MC: Writing – review & editing, Supervision, Resources, Formal analysis. RB: Writing – review & editing, Supervision, Software, Project administration, Funding acquisition. FK: Writing – review & editing, Supervision, Software, Resources, Project administration, Formal analysis, Conceptualization.
